# Different tactics, one goal: initial reproductive investments of males and females in a small Arctic seabird

**DOI:** 10.1007/s00265-014-1761-4

**Published:** 2014-07-04

**Authors:** Katarzyna Wojczulanis-Jakubas, Dariusz Jakubas, Olivier Chastel

**Affiliations:** 1 Department of Vertebrate Ecology and Zoology, University of Gdańsk, ul. Wita Stwosza 59, 80-308 Gdańsk, Poland; 2 Centre d’Etudes Biologiques de Chizé, Centre National de la Recherche Scientifique, Villiers en Bois, France

**Keywords:** *Alle alle*, Little auk (dovekie), Corticosterone, Extra-pair copulations, Pre-laying parental investments

## Abstract

Despite a great number of studies on extra-pair paternity in birds, the actual roles of males and females in extra-pair contacts is poorly understood, as detailed behavioural studies comparing the reproductive performance of the two sexes prior to egg laying are relatively scarce. Here, we investigated mating behaviour (copulations and aggressive interactions), time budget and body condition (size-adjusted body mass and baseline corticosterone level) in the little auk (*Alle alle*), a monogamous and highly colonial, Arctic seabird. We performed the study in a large breeding colony of the little auk in Hornsund (Spitsbergen). We found that the males frequently attempted extra-pair copulations (EPCs), although these contacts were almost always unsuccessful, mostly because of the females’ rejection behaviour. These results clearly indicate that genetic monogamy is maintained through female control. Nevertheless, males tried to protect their paternity by staying in close proximity to their females and aggressively intervening when their mates became involved in EPCs. Compared to females, males also spent more time in the colony guarding nest sites. Despite the apparent sex differences in the time budget and frequency of aggressive interactions, body condition was similar in the two sexes, indicating comparable parental investments during the mating period.

## Introduction

Since Lack’s (1968) study, birds have been viewed as a unique animal group, in which monogamy is the prevailing breeding system. Indeed, a majority of avian males and females form a pair for at least one breeding season, often also caring for the offspring together (Cockburn 2006). However, it has been widely accepted that this avian monogamous system is not free from sexual conflict (Trivers 1972; Clutton-Brock 1991: Birkhead and Møller 1998; Petrie and Kempenaers 1998; Westneat and Stewart 2003; Akçay and Roughgarden 2007). The basic sex differences in the size and number of gametes predispose males and females to different mating strategies. Males, being the ones producing small but numerous spermatozoa should maximise their reproductive success primarily by fertilising as many eggs as possible. Females, in contrast, investing in a limited number of large eggs should aim to mate with a top-quality male. With such divergent male and female reproductive aims, an apparent conflict is expected to arise in socially monogamous pairs (Trivers 1972; Clutton-Brock 1991; Westneat and Stewart 2003). Indeed, a substantial proportion of socially monogamous avian species is sexually promiscuous, with the result that there are >10 % of extra-pair offspring and/or broods (Birkhead and Møller 1998; Petrie and Kempenaers 1998; Westneat and Stewart 2003). On the other hand, there is handful of species in which a very low, if any, extra-pair paternity rate has been reported (e.g. Walsh et al. 2006; Anker-Nilssen et al. 2010; Calderón et al. 2012). Viewing the glass as half empty, one can wonder how genetic monogamy is possible in some birds under conditions of such apparent sexual conflict.

Causality of genetic monogamy is usually provided in the context of ecological constraints and fitness consequences. When conditions are difficult, extensive biparental care is crucial to the successful raising of offspring. In such circumstances, selection should favour a breeding system where both adults are genetically related to the offspring (Trivers 1972; Clutton-Brock 1991). Otherwise, the adult providing care for a non-kin brood decreases its fitness. Indeed, the level of extra-pair brood fertilisation is usually low in species for which biparental care appears to be obligatory (e.g. seabirds; Bennett and Owens 2002; Griffith et al. 2002 but see notable exceptions in Graves et al. 1992; Pilastro et al. 2001).

While genetic relatedness to offspring is usually obvious in the case of females, males may not be certain of their paternity. However, a male may actively guard his paternity by engaging in aggressive interactions with the extra-pair male when the latter is trying to copulate with the female (Birkhead and Møller 1992; Møller and Birkhead 1993). If the male remains in close proximity to the female, he may deter any extra-pair males from approaching the female. The male’s presence may also deter the female from engaging in extra-pair copulations. Although the relationship between the confidence of paternity and the male’s actual care seems to be complex (Wittingham et al. 1992; Sheldon 2002), some studies have shown that males uncertain of their paternity may reduce their parental care (e.g. reviewed in Møller and Birkhead 1993). Thus, if indeed the male’s involvement in parental care is somehow dependent on his certainty of paternity of the brood that he is taking care of, the female should refrain from extra-pair contact in the presence of the social partner (Møller and Birkhead 1993). Finally, if extra-pair copulations have occurred, the male can ensure his paternity through subsequent within-pair copulations, assuming that last-male precedence determines paternity (Birkhead and Møller 1992). What the actual roles of males and females are in maintaining the genetic monogamy remains poorly understood (Westneat and Stewart 2003; Kokko and Jennions 2008), as detailed behavioural studies comparing the reproductive performance of the two sexes prior to egg laying are relatively scarce.

To obtain insight into this issue, we studied the mating interactions of males and females in the little auk (or dovekie, *Alle alle*). We focused on this species for two reasons. First, this is a typical seabird, with both male and female providing long and extensive parental care for a single egg and chick (Harding et al. 2004; Wojczulanis-Jakubas et al. 2009b, 2012). It seems that care by two parents is crucial for raising the offspring successfully (Kidawa et al. 2012). Thus, in the line with the above reasoning, the male’s high investment in parental care should be related to his certainty of paternity. Second, the little auk is a colonially breeding species and the proximity of many conspecifics should facilitate extra-pair mating (Morton et al. 1990; Hunter et al. 1992; Wagner 1992; Møller and Birkhead 1993; but see Griffith et al. 2002). Indeed, little auks have been found to copulate with extra-pair partners quite frequently, although with very limited success (extra-pair paternity in 2 % of investigated families; Lifjeld et al. 2005; Wojczulanis-Jakubas et al. 2009a). However, the mechanisms maintaining genetic monogamy in the little auk are unknown, as mating behaviour of this species has not been studied in detail.

To evaluate the possible mechanisms responsible for the limited success of extra-pair copulations in the little auk, we investigated the pre-laying behaviour (copulations and aggressive interactions) and colony attendance pattern of males and females. Additionally, to establish the costs of these mating behaviours for the two sexes, we measured the morphological and physiological body condition of the birds. To measure morphological condition (considered here as the relative magnitude of energy reserves in the form of fat and proteins, Gosler 1996), we used body mass corrected for body size by calculating the scaled mass index (SMI; Peig and Green 2009, 2010). The SMI standardises all individuals to the same body size, adjusting their body mass to the one they would have at their new body size in accordance with the scaling trend between body mass and body size (Peig and Green 2009, 2010). It has been shown in many bird species, including the little auk that the body mass of birds (appropriately corrected for body size) decreases under conditions of food deprivation and in response to elevated efforts related to parental performance (e.g. Taylor 1994; Moe et al. 2002; Williams et al. 2007; Jakubas et al. 2013). For the physiological condition, we used the baseline corticosterone concentration (CORT). The baseline level of this hormone has been found to be correlated positively with increased parental efforts (e.g. Doody et al. 2008). If there were any differences in effort related to mating performance between male and female, we would expect to find corresponding sex differences in body condition.

## Methods

### Study area and field methods

We conducted the study in a large little auk breeding colony on the Ariekammen slopes in Hornsund (SW Spitsbergen; 77° 00′ N, 15° 33′ E) during the pre-laying period in 2011. To examine the behaviour and body condition (SMI and CORT) of males and females, we captured adult birds 11–15 days prior to the median egg-laying date in the colony. At this time, the birds had been in the colony for ca 8 weeks after the first, post-wintering appearance and their sexual activity was approaching peak level (Wojczulanis-Jakubas et al. 2009a), so body condition parameters could serve as a proxy of the birds’ energetic state related to the mating performance. At the same time, females had not yet started to form eggs (5 days before laying, J. Taylor and M. Konarzewski, unpublished data), as this process could affect both the body mass and hormones levels. We did not know the exact date when most captured birds laid their egg, but given the considerable laying synchrony in the colony, we could assume a similar phase of breeding for all individuals. All eggs were laid within 7 days, with

the majority of eggs (70 %) laid within 3 days in the control group of 68 nests (located in the same area where the target birds were captured; the nests were inspected every day starting from a week before the expected median egg-laying date). Additionally, to confirm the breeding status and phase of the sampled birds, we performed additional observations in the first week after the median egg-laying date (none of the birds were seen copulating).

To minimise disturbance at the colony, caused by capturing the birds, we deployed noose-carpets over a small colony patch (ca 200 m^2^). The use of these noose-carpets allowed us to minimise the time for which the birds were releasing to just a few seconds, which is crucial when the baseline corticosterone level is being measured (Wingfield 1994). Immediately after each bird was captured, we took a blood sample from the brachial vein using a 200-μL heparinised capillary for analysing the corticosterone level and for molecular sexing (sexing according to morphological features is not reliable; Jakubas and Wojczulanis 2007). We timed the duration of blood sampling precisely, starting from moment that the birds were caught in the noose carpet (av. duration = 2.10 ± SD: 0.55 min). There was no correlation between the duration of handling and sampling, and the baseline corticosterone level (Pearson correlation coefficient, *r*_58_ = 0.14, *p* = 0.28). We sampled all the birds within a moderate time window of 9 h. The baseline corticosterone levels were not correlated with the time of day (*r*_58_ < 0.001, *p* = 0.99). We kept the blood cool (+4 °C) for 2–3 h until centrifugation for 10 min at 6,000 rpm. We kept the separated plasma and red cells frozen (at −20 °C) and analysed them within 4 months. We weighed all birds with a Pesola spring scale (±1 g accuracy) and measured their head-bill length. We marked all the birds with unique dyed signs on the breast feathers and a combination of coloured and metal leg rings for further observations. The dyed signs were made with waterproof, permanent markers (*Sharpie*, USA); although the marks faded with time, they were still well visible during the last observation session. We sampled each bird only once. We captured, weighed, measured, blood-sampled and marked 68 birds but in the case of nine individuals, the amount of blood taken was insufficient for analysing the corticosterone level. In all, therefore, we collected data for hormone level analyses from 27 males and 32 females.

We observed the marked individuals (two observers simultaneously, the same two for all observation sessions) for at least 5 h each day (between 0900 and 1900 hours as the birds were mostly present in the colony during that time; Wojczulanis-Jakubas et al. 2009a) starting from the 10th day prior to laying until the day of the first record of an egg in the control nests (3 days prior to the median egg-laying date for the whole colony). We observed the birds for a total of 54.2 h. Of the 68 marked birds, 36 males and 30 females (including 18 social pairs with both partners marked) turned out to occupy a nest site within the capture area, in close proximity to one another. The two birds that occupied territories outside the capture area were not considered in the behavioural analyses. Each of 66 marked birds was observed for on average of 14.2 ± SD: 8.9 h throughout the observation period. Of these 66 birds, 26 males and 24 females were sampled for analysis of corticosterone level.

During each hour of observation, we recorded the presence/absence of individually marked birds in the nest site area. We observed the birds continuously and noted their presence every 10 min. For each bird, we also noted the frequency of copulations and the aggressive interactions they were involved in. We classified the copulations of social and non-social mates as within-pair (WPC) and extra-pair (EPC) copulations, respectively. We considered a copulation to be successful when the male mounted the female with both feet placed on her back, moved his tail from side to side and achieved at least one cloacal contact. In cases where it was not possible to see directly whether cloacal contact had been achieved, we used mounting duration and female behaviour as an indicator of copulation success. Unsuccessful copulations were short, often without female cooperation (she raised her body into an upright position, thereby preventing the male from sitting on her back, Wojczulanis-Jakubas et al. 2009a). For each record of aggressive interaction, we distinguished the initiator and recipient of the interaction as well as its intensity. We considered the initiator to be the bird that was the first to behave agonistically. We discriminated three degrees of intensity of aggressive interactions: (1) threatening [birds taking up a threatening posture (bill open and/or feathers bristling and/or wings/head lowered) but without observed physical contact], (2) threatening with physical contact (besides the threatening, brief physical contact took place) and (3) fight (longer and intensive physical contact).

### Laboratory analyses

We measured baseline levels of total (free and bound) corticosterone by radioimmunoassay. We measured the total concentration after ethyl ether extraction using a commercial antiserum, raised in rabbits against corticosterone-3-(O-carboxymethyl) oxime bovine serum albumin conjugate (Biogenesis, UK). Cross reaction was 9 % with 1-desoxycorticosterone and less than 0.1 % with other plasma steroids. We incubated duplicate aliquots (100 μl) of the extracts overnight at 4 °C with 8,000 cpm of 3H-Corticosterone (Amersham Pharmacia Biotech-France) and antiserum. We separated the free and bound fractions of corticosterone by adding dextran-coated charcoal. After centrifugation, we counted the bound fraction in a liquid scintillation counter. Minimal detectable corticosterone levels were 0.3 ng. To measure intra-assay variation, we included four different samples ten times in the corticosterone assay. From this, the intra-assay variation for total corticosterone was the 6.7 % (range, 5–12 %).

We extracted DNA for sexing from the frozen blood cells using a Blood Mini kit (A&A Biotechnology, Gdynia, Poland). We performed CHD gene-based analyses with the primer pair F2550 and R2718, according to Griffiths et al. (1998), using a 50 °C annealing temperature for the polymerase chain reaction (PCR). The sex differences in the PCR products were clearly visible in UV light when we separated the fragments on 2 % agarose gel stained in ethidium bromide.

### Statistical analyses

Since we observed the birds for a variable amount of time on the consecutive days of the pre-laying period, we calculated the standardised time spent by each individual in the colony on particular days. For that purpose, we divided the total time the bird was recorded at the nest site area by the duration of the observation session on a given day. We analysed the total time and the time spent in the colony without the partner (both standardised) during the consecutive days of the pre-laying period using factorial ANOVA in the mode of linear mixed models, with sex and date as fixed factors. We also included in the model the interaction between the two variables. As the same individuals were observed during the consecutive days of the pre-laying period, we included in the model the birds’ identity as random factor to avoid the problem of pseudoreplication. We used unequal-N HSD as a post hoc test for significant differences.

We calculated the frequency of copulations (WPCs and EPCs separately) and aggressive interactions (both separately and jointly for all degrees) per hour of time spent in the colony by each target bird. As WPCs could serve as mechanisms preventing extra-pair fertilisations (Birkhead et al. 1985, 1987), we compared the number of female EPCs with the rate of WPCs using simple Pearson correlation analysis. Using the same line of reasoning, we used 2 × 2 Chi-square test of association to compare the proportion of female EPCs followed and not followed by WPC (within a 20-min time frame). To further assess the performance of extra-pair contacts, we used 2 × 2 Chi-square test of association to compare the proportion of EPCs occurrence in relation to the presence/absence of their partners. We also used the 2 × 2 Chi-square test of association to compare the proportion of occurrence male aggressive interventions at the moment of female EPC. To check whether the frequency of EPC attempts in females is related to the social status of males, we compared the number of female EPCs with the total number of aggressive interactions, the number of interactions initiated and received by the male, using Pearson’s correlations. Finally, we compared the frequency of aggressive behaviour between the sexes with the Mann-Whitney *U* tests.

To analyse a bird’s body mass, we used the SMI. We computed the SMI using the formula (Peig and Green 2009):

$$ SMI= Mi{\left[\frac{Lo}{Li}\right]}^{bSMA} $$

where *M*_*i*_ is the body mass of individual *i*; *L*_*i*_ is the linear body measurement of individual *i* (overall head length) and *b*_*SMA*_ is the scalling exponent estimated from the regression of *M* and *L. Lo* is the arithmetic mean value of the linear measurement. We used the mean value of overall head length for the target population, as this measurement was significantly correlated with the body mass (*r*_68_ = 0.60 , *p* < 0.001). We calculated the scaling exponent by dividing the slope of the ordinary linear square regression of *lnM* and *lnL* by the Pearson’s correlation coefficient (LaBarbera 1989; Peig and Green 2009). We compared the SMI between the sexes using Student’s *t* test. We also used this test to compare corticosterone concentrations between males and females. To check how the copulation and aggressive behaviours relate to bird’s body condition, we performed a Pearson correlation of the total number of copulations and aggressive interactions with SMI and CORT, separately for males and females.

We analysed the birds’ behaviour using two data sets: the first combined all marked birds (36 males and 30 females), and the second combined only pairs with both partners marked (18 pairs). We used the first set of data for general comparisons of male and female behaviour and the second one whenever within-pair interactions (WPC, EPC, time spent in the colony with and without the partner) were considered. We checked the assumptions of normality and homogeneity of variance of all variables with the Shapiro-Wilk and Levene tests, respectively. We used parametric analyses and provided parametric statistics (mean ± standard error, SE) when the assumptions were met; otherwise, we used the non-parametric tests and provided non-parametric statistics (median and 25–75 % quartiles). We performed all the analyses in STATISTICA 9.1 (Statsoft Inc.) and SPSS 21 (IBM Corp.). We considered the value of *p* = 0.05 the threshold for significant differences.

### Ethical note

All birds were ringed on the basis of licence no 1095 and handled with permission from the Norwegian Animal Research Authority and the Governor of Svalbard (2011/00150-18). Blood sampling did not appear to have any detrimental effect on the handled birds. All of them were released unharmed after ca 10 min of handling. The presence of observers and the colour marks on the breast feathers did not seem to influence the birds’ behaviour as all of them behaved normally. The artificial marks faded away with time; 4 weeks after marking, the signs were hardly visible.

## Results

### Time spent in the colony

**Fig. 1:**
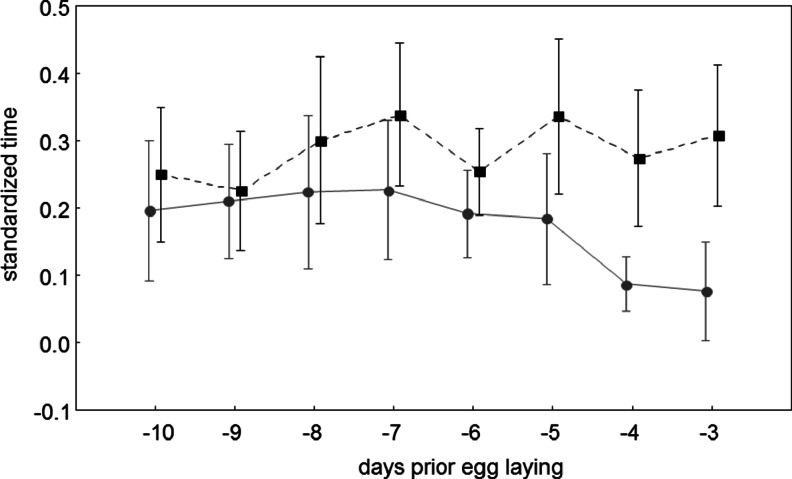
The total standardised time (the total time during which the bird was recorded in the nest site area divided by the duration of the observation session on a given day; means with 95 % confidence interval) spent in the colony by marked males (black squares) and females (*grey circles*) during the pre-laying period

The males spent significantly more time in the colony than the females (ANOVA, *F*_1,34_ = 7.50, *p* = 0.01). The total time spent by the birds in the colony was similar throughout the whole study period (*F*_7,238_ = 1.87, *p* = 0.08). Although females appeared to be present in the colony less frequently as the laying date was approaching (Fig. 1), there was no significant interaction in the time spent in the colony between the sex of birds and the day of the pre-laying period (*F*_7,238_ = 1.95 *p* = 0.06).

**Fig. 2:**
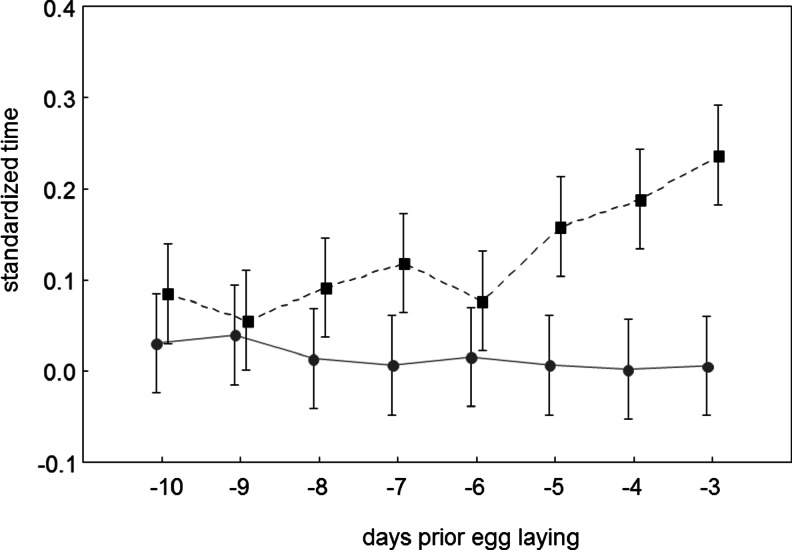
The standardised time (the total time during which the bird was recorded in the nest site area divided by the duration of the observation session on a given day; means with 95 % confidence interval) spent in the colony without the partner during the pre-laying period (males - *black squares*; females - *grey circles*)

In contrast to the males, the females were rarely present in the colony when their mates were absent (ANOVA, *F*_1,34_ = 22.91, *p* < 0.001; Fig. 2). There was significant differences in the time spent in the colony among the particular days of the pre-laying period (*F*_7,238_ = 2.53, *p* = 0.02; Fig. 2), with significant interaction between sex and day of the pre-laying period (*F*_7,238_ = 4.78, *p* < 0.001). Sex differences were particularly obvious when the birds were approaching the egg-laying period (unequal-N HSD test, Table 1, Fig. 2), with males spending more time in the colony without their partners in the last days of the pre-laying period.

**Table 1:** Results of unequal-N HSD test (*P* values) for the interaction of sex and day of pre-laying period in the analysis of influence of these two variables on the standardised time spent by the little auks in the colony without their social partner

	Day prior laying	-10	-9	-8	-7	-6	-5	-4	-3	-10	-9	-8	-7	-6	-5	-4
Day prior laying	Sex	F	F	F	F	F	F	F	F	M	M	M	M	M	M	M
-10	F															
-9	F	1.00														
-8	F	1.00	1.00													
-7	F	1.00	1.00	1.00												
-6	F	1.00	1.00	1.00	1.00											
-5	F	1.00	1.00	1.00	1.00	1.00										
-4	F	1.00	1.00	1.00	1.00	1.00	1.00									
-3	F	1.00	1.00	1.00	1.00	1.00	1.00	1.00								
-10	M	1.00	1.00	0.92	0.83	0.93	0.83	0.76	0.82							
-9	M	1.00	1.00	1.00	1.00	1.00	0.98	1.00	1.00	1.00						
-8	M	0.98	1.00	0.84	0.72	0.86	0.72	0.63	0.70	1.00	1.00					
-7	M	0.67	0.82	0.35	0.23	0.38	0.24	0.18	0.22	1.00	0.97	1.00				
-6	M	1.00	1.00	0.97	0.91	0.97	0.92	0.86	0.91	1.00	1.00	1.00	1.00			
-5	M	0.08	0.15	**0.02**	**0.01**	**0.02**	**0.01**	**0.01**	**0.01**	0.88	0.37	0.94	1.00	0.78		
-4	M	**0.005**	**0.013**	**<0.001**	**<0.001**	**0.001**	**<0.001**	**<0.001**	**<0.001**	0.35	0.05	0.48	0.92	0.24	1.00	
-3	M	**<0.001**	**<0.001**	**<0.001**	**<0.001**	**<0.001**	**<0.001**	**<0.001**	**<0.001**	**0.01**	**<0.001**	**0.02**	0.16	**0.01**	0.83	1.00

Significant values in bold

### Copulations and aggressive interactions

Almost half (43 %) of 437 recorded WPCs were successful (involved cloacal contact), with on average 1.2 (±0.61) successful WPCs per hour of both partners staying together in the colony. Most EPCs were unsuccessful. In fact, only 1 (2 %) of the 49 EPC events recorded was considered to be successful. All EPCs were initiated by males and the female’s rejection behaviour (she raised her body into an upright position, thereby preventing contact with the male’s cloaca) was the prime reason for the low success rate. Most (64 %) of 38 marked individuals (50 % males and 78 % females) were involved in EPCs. In these birds, EPCs made up 16.1 ± 14.34 % of all copulation attempts in males and 14.9 ± 14.41 % in females. The numbers of WPCs and EPCs were not significantly correlated in females (*r*_17_ = 0.35, 1.59, *p* = 0.13).

The majority of EPC attempts (85 %, *n* = 19) initiated by the marked males occurred when their partners were away from the colony (*χ* ^2^ test, *χ* ^2^_1_ = 5.51, *p* = 0.02). In contrast, the majority of EPC attempts (97 %, *n* = 30) received by the marked females occurred when their mates were present in the colony (*χ* ^2^_1_ = 17.28, *p* < 0.001). The frequency of WPCs following a female EPC attempt was low. Only 24 % of EPC attempts performed in the presence of the partners were followed by WPC (*χ* ^2^_1_ = 5.33, *p* = 0.02). However, the male partners usually intervened aggressively (with second or third degree intensity) during the EPC attempt of their females (89 % of events with male intervention of second and third degree aggressiveness; *χ* ^2^_1_ = 6.87, *p* = 0.01).

The total number of aggressive interactions was higher in males than in females (Table 2). This sex difference was also apparent when considering the aggressive interactions of each degree of intensity separately (Table 2). Also, males initiated aggressive interactions more frequently than females (Table 2). However, the number of interactions in which the target bird acted as recipient was similar in both sexes (Table 2). The identity of the partner in the aggressive interaction with the focal bird was usually unknown. However, based on the records of interactions between the individuals of known identity (*n* = 107), males and females initiated/received aggressive interactions with/from both the same and opposite sex. Aggressive interactions between the breeding pair members has never been observed. The number of EPCs in females was not related to the total number of aggressive interactions in which her mate was involved (*r*_17_ = 0.39, *p* = 0.11), or to the number of aggressive interactions initiated by him (*r*_17_ = 0.18, *p* = 0.49). There was a tendency toward a positive relationship between the number of EPC attempts in females and overall number of aggressive interactions received by her partner (*r*_17_ = 0.47, *p* = 0.06).

**Table 2:** Sex differences in the number of aggressive interactions of different categories and role of the birds in these interactions (Mann-Whitney *U* tests)

Category of interaction (number/h)	*Z*	*p*	Females (*n* = 27)				Males (*n* = 32)			
			Median	*Q*1	*Q*3	Range (min–max)	Median	*Q*1	*Q*3	Range (min–max)
Type of aggressive interactions:										
All	−5.70	<0.001	0.38	0.2	0.78	0.00–1.75	1.90	2.81	1.31	0.17–4.45
Threatening (1st degree)	−4.56	<0.001	0.16	<0.001	0.26	0.00–1.33	0.72	0.97	0.43	0.00–1.65
Threatening with physical contact (2nd degree)	−4.58	<0.001	0.17	<0.001	0.30	0.00–1.50	0.83	1.38	0.47	0.00–2.78
Fight (3rd degree)	−5.67	<0.001	<0.001	<0.001	0.00	0.00–0.41	0.28	0.50	0.23	0.00–1.8
Role in aggressive interactions:										
Receiver	−1.73	0.08	0.22	0.08	0.55	0.00–1.33	0.34	0.57	0.21	0.00–1.10
Initiator	−6.28	<0.001	0.00	<0.001	0.06	0.00–0.16	0.76	0.38	1.19	0.00–1.9

### Body condition parameters

No significant sex difference was found in the SMI (males, 161.83 ± 9.42; females, 163.74 ± 10.41; *t* test, *t*_66_ = 0.79, *p* = 0.43)*.* Also, baseline corticosterone levels (CORT) were similar in males (mean, 5.03 ± 0.54 ng/mL, *n* = 27) and females (mean, 4.37 ± 0.54 ng/mL, *n* = 32; Student’s *t* test, *t*_57_ = −0.87, *p* = 0.39). There were no correlations between SMI and the total number of copulations in both males (*r*_35_ = −0.17, *p* = 0.50) and females (*r*_29_ = −0.02, *p* = 0.94). Moreover, in neither sex was SMI related to the total number of aggressive interactions (males: *r*_35_ = −0.31, *p* = 0.21; females: *r*_29_ = −0.01; all *p* = 0.96). CORT was not related to total number of copulations in females (*r*_23_ = −0.22, *p* = 0.37) but there was a tendency towards a negative correlation in males (*r*_25_ = −0.43, *p* = 0.09). Similarly, CORT was not related to the total number of aggressive interactions in females (*r*_23_ = −0.19, *p* = 0.46) but there was a tendency towards a negative correlation in males (*r*_25_ = −0.46, *p* = 0.07).

## Discussion

The rate of EPC attempts reported in this study (10 % of all recorded copulations were extra-pair contacts) and in the previous one performed in the same colony (24 %, Wojczulanis-Jakubas et al. 2009a) is similar to that reported in other alcid, closely related to the little auk and also colonially breeding, the common guillemot (*Uria aalge*) (12 %, Walsh et al. 2006). Such rates of EPC events seem to be high and could be related to the colonial breeding. In solitary breeders, for example, the American kestrel (*Falco sparverius*) extra-pair contacts occur with very low frequency (<1 %; Villaroel et al. 1998). However, in other, colonially breeding seabirds such as the king penguin (*Aptenodytes patagonicus*) and the northern fulmar (*Fulmarus glacialis*), the rate of extra-pair contacts did not exceed 3 % of all copulations observed (Hunter et al. 1992; Olsson et al. 2001). Therefore, factors other than nest density, possibly specific for group/species, may be responsible for the high rate of extra-pair copulation attempts. This highlights the suitability of the little auk as a species for studying sex differences in mating strategy.

Despite the high frequency of EPC attempts, hardly any of these contacts were successful. Little auk females seem to avoid insemination during forced EPCs by standing up and preventing cloacal contacts. This is consistent with a previous study assessing the effectiveness of EPCs in the little auk (Wojczulanis-Jakubas et al. 2009a). It is, however, in contrast with other closely related auks [14 % of successful EPCs in the razorbill (*Alca torda*), Wagner 1991; 32 % in the common guillemot, Walsh et al. 2006]. These interspecies differences may be related to the females’ behaviour. A higher proportion of successful EPCs were initiated by razorbill and common guillemot females (Wagner 1991; Walsh et al. 2006), whereas females in the present study appeared not to initiate EPCs at all. Why the little auk females are so reluctant to EPCs is difficult to explain. However, all these results of alcids behaviour indicate that males forcing extra-pair copulations cannot properly mount without the female’s cooperation. This, in turn, corroborates that avian females are capable of controlling extra-pair fertilisation in some species. This female control may happen at different stages of the breeding cycle: before copulation; during copulation; after copulation but before fertilisation and following fertilisation (e.g. Wagner 1991; Birkhead and Møller 1993; Graves et al. 1993; Wagner et al. 2004; Adler 2010; Brekke et al. 2013). The behavioural control observed in the mentioned auks, including the little auk, is probably the earliest possible and most appropriate in colonial breeding conditions.

Although the effectiveness of EPCs seemed to be under female control, little auk males intervened aggressively when their females were a subject of EPC attempt. This was also the case in some of studies from the common guillemot (Birkhead et al. 1985; Hatchwell 1988). Given this female rejection behaviour, the risk of cuckoldry seems to be low in both species. Nevertheless, the males appeared to actively guard their paternity. This clearly shows that males need assurance of their paternity in the social pair based on their own control.

Frequent WPCs may also play a role in ensuring the male about his paternity. This could work not necessarily through the mechanism of last-male precedence as proposed for other species (Birkhead et al. 1987; Birkhead and Møller 1992), but indirectly, through increasing the males certainty of his paternity in the social pair. Although no direct relationship between the occurrence and number of WPCs after EPCs was found in the present study, quite high number of EPCs (24 %) were followed by WPC. Moreover, the frequency of successful WPCs (on average 1.2 per hour of both partners present in the colony), during the whole pre-laying period was far higher than necessary to fertilise the single egg in the clutch.

Little auk females were usually accompanied by their partner while in the colony. This high proportion of co-attendance might be an additional mechanism for guarding paternity. If the male’s involvement in parental care is somehow dependent on his certainty of paternity of the brood that he is caring for, the female should not risk losing his contribution (e.g. Wittingham et al. 1992; Møller and Birkhead 1993; Westneat and Sargent 1996; Sheldon and Ellegren 1998). Kidawa et al. (2012) showed that reduced care provided by one little auk parent (due to the GPS loggers attachment) noticeably lowered breeding success of the pair. Hence, little auk females may refrain from EPCs to prevent potential loss of their partner’s contribution to parental care.

Males pursued EPCs mainly while their mates were away from the colony. This pursuit of EPCs by males suggests that their prolonged stay in the colony during the pre-laying period may be at least partly related to an extra-pair mating opportunity. EPCs are rarely successful in the little auk due to the female reluctant behaviour (Wojczulanis-Jakubas et al. 2009a; this study), but some EPCs can apparently result in extra-pair fertilisation (2 %, Wojczulanis-Jakubas et al. 2009a). For the guillemots, in which females also seem to control success of extra-pair copulation, females accepted EPCs when they have not yet been reunited with their own partner and/or in the process of switching mates (Walsh et al. 2006). If that is also the case in the little auk, there is still a good chance for the males to achieve extra-pair paternity. Once the egg is fertilised, breeding success is quite high (Jakubas and Wojczulanis-Jakubas 2011). Thus, having a single extra-pair offspring may double the male’s breeding success at a given breeding attempt. Alternatively, males’ prolonged staying in the colony could be related to nest site guarding. At all times in the colony, males were in close proximity to their nest, frequently involving into aggressive interactions. This suggests strong competition for nest sites, and so males’ role in maintenance of the nest territory.

These initial activities of little auk males may be viewed as costly. Firstly, the time spent by males in the colony reduces the time available for foraging. Secondly, staying in the colony requires constantly focused attention by virtue of predator pressure (Stempniewicz 1995; Wojczulanis et al. 2005). The probability of being predated is likely to be higher in the colony than at the sea, owing to the occurrence of a terrestrial predator (the Arctic fox *Vulpes lagopus*) and to the smaller predative capability of the glaucous gull *Larus hyperboreus* at the sea (no possibility to pursue diving little auk). Moreover, the birds are scared away by the appearance of the predator in the colony several times per hour, which may be energy-consuming (Wojczulanis et al. 2005). Finally, frequent involvement of the males in aggressive interactions costs them time and energy and may additionally increase the risk of their being predated.

Females spent much of their time away from the colony, probably foraging for the purpose of the egg formation, as this is the case in other seabirds (Astheimer et al. 1985; Creelman and Storey 1991). Also, females were involved in aggressive interactions less often than males and hardly ever initiated them. Given these results, one might expect a higher body mass and a lower corticosterone levels in females than in males. However, both the scaled mass index and the baseline corticosterone level were similar in the two sexes. This is in line with the results from another alcid, the Atlantic puffin *Fratercula arctica*, where no significant sex differences in body mass and CORT level were found at any individual breeding stage, including the pre-laying period (although females had higher CORT levels overall; Rector et al. 2012). This similarity of body condition in little auk males and females indicates similar parental efforts during the pre-laying period. This further suggests that the female’s initial investments, although quite different in nature, are at least as costly as the male’s pre-laying activity. Obviously, we cannot rule out the possibility that the pattern of the sex differences in body condition will be different at the very end of the pre-laying period, after the females have completed egg-laying. In fact, we report the birds body condition at a time when this crucial female investment has not yet been made. Also, we did not find correlations between SMI and CORT and the total number of copulations and aggressive interactions, although there was a tendency for a negative correlations in males. Therefore, further studies would be desirable to assess the energy budget of the sexes throughout the whole initial stage of breeding.

Finally, it is worth pointing out that we found a tendency toward a positive relationship between the number of EPC attempts directed towards females and the number of aggressive interactions received by their partners. The relationship was close to significant (*p* = 0.06). This result is interesting as it suggests existence of social hierarchy in the little auk colony, which actually has once been proposed (Kharitonov 2007). So, female’s involvement in EPCs might depend on her own social status, the status of her partner and/or the pair. Further studies will be necessary to evaluate this relationship.

Summing up, the results of the present study confirmed the basic difference between male and female mating strategies. No matter what the breeding system is, the male pursues extra-pair contacts while the female carefully chooses her sexual partner. The results also clearly indicate that the genetic monogamy is maintained through female control. However, determining the rules underlying the female’s choice requires further investigation.

## References

[CR1] Adler M (2010). Sexual conflict in waterfowl: why do females resist extrapair copulations?. Behav Ecol.

[CR2] Akçay E, Roughgarden J (2007). Extra-pair parentage: a new theory based on transactions in a cooperative game. Evol Ecol Res.

[CR3] Anker-Nilssen T, Kleven O, Aarvak T, Lifjeld JT (2010). Low or no occurrence of extra-pair paternity in the Black Guillemot *Cepphus grylle*. J Ornithol.

[CR4] Astheimer LB, Price PA, Grau CR (1985). Egg formation and the pre-laying period of Black-browed and Grey-headed Albatrosses *Diomedea melanophris and D. chrysostoma* at Bird Island, South Georgia. Ibis.

[CR5] Bennett PM, Owens IPF (2002). Evolutionary ecology of birds: life history, mating systems and extinction.

[CR6] Birkhead TR, Atkin L, Møller AP (1987). Copulation behaviour of birds. Behaviour.

[CR7] Birkhead TR, Johnson SD, Nettleship DN (1985). Extra-pair mating and mate guarding in the common murre *Uria aalge*. Anim Behav.

[CR8] Birkhead TR, Møller AP (1992). Sperm competition in birds: evolutionary causes and consequences.

[CR9] Birkhead TR, Møller AP (1993). Female control of paternity. Trends Ecol Evol.

[CR10] Birkhead TR, Møller AP (1998). Sperm competition and sexual selection.

[CR11] Brekke P, Cassey P, Ariani C, Ewen JG (2013). Evolution of extreme-mating behaviour: patterns of extrapair paternity in a species with forced extrapair copulation. Behav Ecol Sociobiol.

[CR12] Calderón L, Svagelj WS, Quintana F, Lougheed SC, Tubaro PL (2012). No evidence of extra-pair paternity or intraspecific brood parasitism in the Imperial Shag Phalacrocorax atriceps. J Ornithol.

[CR13] Clutton-Brock TH (1991). The evolution of parental care.

[CR14] Cockburn A (2006). Prevalence of different modes of parental care in birds. Proc R Soc Lond B.

[CR15] Creelman E, Storey AE (1991). Sex differences in reproductive behavior of Atlantic Puffins. Condor.

[CR16] Doody LM, Wilhelm SI, McKay DW, Walsh CJ, Storey AE (2008). The effects of variable foraging conditions on common murre (*Uria aalge*) corticosterone concentrations and parental provisioning. Horm Behav.

[CR17] Gosler AG (1996). Environmental and social determinants of winter fat storage in the great tit *Parus major*. J Anim Ecol.

[CR18] Graves J, Hay L, Scallan M, Rowe S (1992). Extra-pair paternity in the shag, *Phalacrocorax aristotelis*, as determined by DNA fingerprinting. J Zool.

[CR19] Graves J, Ortega-Ruano J, Slater PJB (1993). Extra-pair copulations and paternity in shags: do females choose better males?. Proc R Soc Lond B.

[CR20] Griffiths R, Double MC, Orr K, Dawson RJG (1998). A DNA test to sex most birds. Mol Ecol.

[CR21] Griffith SC, Owens IPF, Thuman KA (2002). Extra pair paternity in birds: a review of interspecific variation and adaptive function. Mol Ecol.

[CR22] Harding AMA, Pelt TIV, Lifjeld JT, Mehlum F (2004). Sex differences in Little Auk *Alle alle* parental care: transition from biparental to parental-only care. Ibis.

[CR23] Hatchwell BJ (1988). Intraspecific variation in extra-pair copulation and mate defence in common guillemots Uria aalge. Behaviour.

[CR24] Hunter FM, Burke T, Watts SE (1992). Frequent copulation as a method of paternity assurance in the northern fulmar. Anim Behav.

[CR25] Jakubas D, Wojczulanis K (2007). Predicting the Sex of Dovekies by Discriminant Analysis. Waterbirds.

[CR26] Jakubas D, Wojczulanis-Jakubas K (2011). Subcolony variation in phenology and breeding parameters in little auk *Alle alle*. Polar Biol.

[CR27] Jakubas D, Wojczulanis-Jakubas K, Kulaszewicz I (2013). Factors affecting haematological variables and body mass of reed warblers (*Acrocephalus scirpaceus*) and sedge warblers (*A. schoenobenus*). Ann Zool Fenn.

[CR28] Kharitonov SP (2007). Methods and theoretical aspects of seabird studies.

[CR29] Kidawa D, Jakubas D, Wojczulanis-Jakubas K, Iliszko L, Stempniewicz L (2012). The effects of loggers on the foraging effort and chick-rearing ability of parent little auks. Polar Biol.

[CR30] Kokko H, Jennions MD (2008). Parental investment, sexual selection and sex ratios. J Evol Biol.

[CR31] LaBarbera M (1989). Analyzing body size as a factor in ecology and evolution. Ann Rev Ecol Evol Syst.

[CR32] Lack D (1968). Ecological adaptations for breeding in birds.

[CR33] Lifjeld JT, Harding AMA, Mehlum F, Øigarden T (2005). No evidence of extra-pair paternity in the little auk *Alle alle*. J Avian Biol.

[CR34] Moe B, Langseth I, Fyhn M, Gabrielsen GW, Bech C (2002). Changes in body condition in breeding kittiwakes *Rissa tridactyla*. J Avian Biol.

[CR35] Møller AP, Birkhead TR (1993). Certainty of paternity covaries with paternal care in birds. Behav Ecol Sociobiol.

[CR36] Morton ES, Forman L, Braun M (1990). Extra-pair fertilization and the evolution of colonial breeding in purple martin. Auk.

[CR37] Olsson O, Bonnedahl J, Anker-Nilssen P (2001). Mate switching and copulation behaviour in King Penguin. J Avian Biol.

[CR38] Peig J, Green AJ (2009). New perspective for estimating body condition from mass/length data: the scaled mass index as an alternative method. Oikos.

[CR39] Peig J, Green AJ (2010). The paradigm of body condition: a critical reappraisal of current methods based on mass and length. Funct Ecol.

[CR40] Petrie M, Kempenaers B (1998). Extra-pair paternity in birds: explaining variation between species and populations. Trends Ecol Evol.

[CR41] Pilastro A, Pezzo F, Olmastroni S, Callegarin C, Corsolini S, Focardi S (2001). Extrapair paternity in the Adélie Penguin *Pygoscelis adeliae*. Ibis.

[CR42] Rector ME, Kouwenberg A-L, Wilhelm SI, Robertson GJ, McKay DW, Fitzsimmons MG, Baker CR, Cameron-MacMillan ML, Walsh CJ, Storey AE (2012). Corticosterone levels of Atlantic puffins vary with breeding stage and sex but are not elevated in poor foraging years. Gen Comp Endocrinol.

[CR43] Sheldon BC (2002). Relating paternity to paternal care. Phil Trans R Soc B.

[CR44] Sheldon BC, Ellegren H (1998). Paternal effort related to experimentally manipulated paternity of male collared flycatchers. Proc R Soc Lond B.

[CR45] Stempniewicz L (1995). Predator–prey interactions between glaucous gull *Larus hyperboreus* and little auk *Alle alle* in Spitsbergen. Acta Ornithol.

[CR46] Taylor JRE (1994). Changes in body mass and body reserves of breeding little auks (*Alle alle* L.). Polish. Polar Res.

[CR47] Trivers RL, Campbell B (1972). Parental investment and sexual selection. Sexual selection and the descent of man.

[CR48] Villaroel M, Bird DM, Kuhnlein D (1998). Copulatory behaviour and paternity in the American kestrel: the adaptive significance of frequent copulations. Anim Behav.

[CR49] Wagner R (1991). Evidence that female razorbills control extra-pair copulations. Behaviour.

[CR50] Wagner R (1992). Extra-pair copulations in lek: the secondary mating system of monogamous razorbills. Behav Ecol Sociobiol.

[CR51] Wagner RH, Helfenstein F, Danchin E (2004). Female choice of young sperm in a genetically monogamous bird. Proc R Soc Lond B.

[CR52] Walsh CJ, Wilhelm SI, Cameron-MacMillan ML, Storey AE (2006). Extra-pair copulations in common murres I: A mate attraction strategy?. Behaviour.

[CR53] Westneat DF, Sargent RC (1996). Sex and parenting: the effects of sexual conflict and parentage on parental strategies. Trends Ecol Evol.

[CR54] Westneat DF, Stewart IRK (2003). Extra-pair paternity in birds: causes, correlates, and conflict. Ann Rev Ecol Evol Syst.

[CR55] Williams CT, Kildaw SD, Buck CL (2007). Sex-specific differences in body condition indices and seasonal mass loss in tufted puffins. J Field Ornithol.

[CR56] Wingfield JC, Davey KG, Peter RE, Tobe SS (1994). Modulation of the adrenocortical response in birds. Perspectives in comparative endocrinology.

[CR57] Wittingham LA, Taylor DP, Robertson RJ (1992). Confidence of paternity and male parental care. Am Nat.

[CR58] Wojczulanis-Jakubas K, Jakubas D, Kidawa D, Kośmicka A (2012). Is the transition from biparental to male-only care in a monogamous seabird related to changes in body mass and stress level?. J Ornithol.

[CR59] Wojczulanis-Jakubas K, Jakubas D, Øigarden T, Lifjeld JT (2009). Extra-pair copulations are frequent but unsuccessful in a highly colonial seabird, the little auk, *Alle alle*. Anim Behav.

[CR60] Wojczulanis K, Jakubas D, Stempniewicz L (2005). Changes in the glaucous gull predatory pressure on little auks in southern Spitsbergen. Waterbirds.

[CR61] Wojczulanis-Jakubas K, Jakubas D, Stempniewicz L (2009). Sex-specific parental care by incubating Little Auks (*Alle alle*). Ornis Fenn.

